# GUI development for SSDL to calibrate photon measuring equipment

**DOI:** 10.1016/j.mex.2023.102408

**Published:** 2023-10-06

**Authors:** Omaima Essaad Belhaj, Siham Belhaj, Meryeme Bellahsaouia, Younes Sadeq, Maryam Hadouachi, Khaoula Laazouzi, Assia Arectout, Hamid Boukhal, Chakir El mahjoub, Tahar El Khoukhi

**Affiliations:** a ERSN, Faculty of Sciences, Abdelmalek Essaadi University, Tetouan, Morocco; b ENA, Marrakech, Morocco; c CNRP, Ministry of Health, Sale, Morocco; d LPMS, Faculty of Sciences, Ibn Tofail University, Kenitra, Morocco; e National Center for Energy, Science and Nuclear Technology (CNETSEN), Morocco

**Keywords:** Radiation protection, Calibration factor, ISO 4037, GUI, H*(10), Python, Development and Validation of a Python-Based Graphical User Interface for Photons Measuring Instruments Calibration at SSDL in Morocco

## Abstract

Current legislation mandates the inspection and calibration of operational survey radiation monitoring instruments used in nuclear medicine, radiotherapy departments, and other fields utilizing ionizing radiation sources. To comply with national and international radiation protection standards, Morocco's National Secondary Standard Dosimetry Laboratory provides reliable calibration results with high accuracy and covers various measurement ranges using attenuators provided by the automated Gamma G10 irradiator or validated beam qualities produced by the X-ray irradiator type X80–320 kV.

This study aims to develop a digital graphical user interface using Python programming language, designed for calibrating radiation protection measuring instruments . The interface is intended to facilitate all operations and calculations related to determining calibration factors and measurement uncertainties in accordance with the ISO 4037 standard, ensuring minimal processing time and minimizing potential error sources . The interface enables calculations to be recorded, as well as the establishment and electronic archiving of the calibration certificate and the report in PDF format using the Hypertext Preprocessor FPDF library (PHP FPDF). With the development of this interface, multiple instruments can be processed per day with high accuracy, streamlining the calibration process and improving efficiency.

• The importance of compliance with international standards to ensure the quality and reliability of measurements in radiation protection was examined.
• Description of X-ray and Gamma-ray irradiators designed for the calibration of radiation protection measuring instruments within the Secondary Dosimetry Calibration Laboratory (SSDL) which is a member of the WHO/IAEA network within the National Center for Radiation Protection of Morocco
• Graphical User Interface using python for the calibration of photon measurement instruments for radiation protection purposes was developped.

## Graphical abstract

**Figure:**
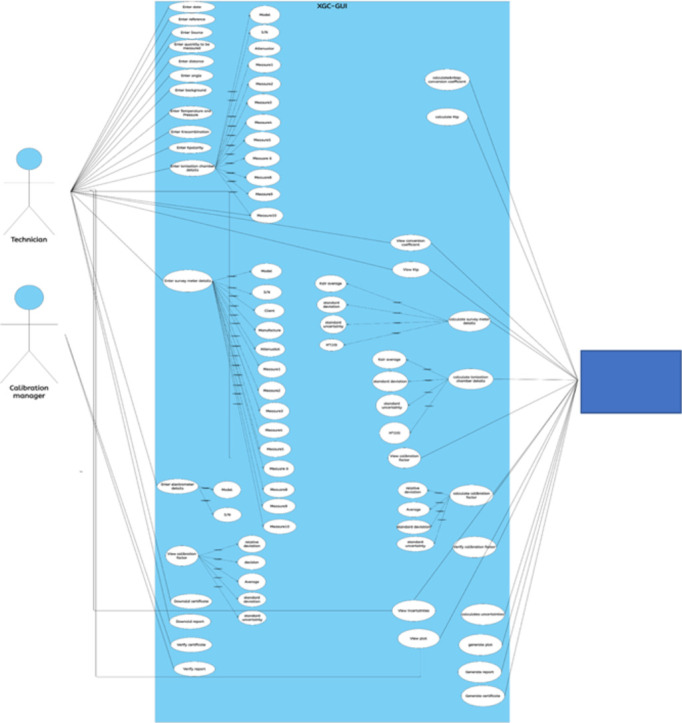

Specification table

**Table:** 

Subject area	Radiation Protection
More specific subject area	Radiation Measurement
Name of your method	Development and Validation of a Python-Based Graphical User Interface for Photons Measuring Instruments Calibration at SSDL in Morocco.
Name and reference of original method	Calibration of radiation protection monitoring instruments”, Safety Reports Series No 16, IAEA, Vienna, Austria (2000) [1]; Radiological protection-X and gamma reference radiation for calibrating dosemeters and doserate meters and for determining their response as a function of photon energy part 3: calibration of area and individual dosimeters and measurement of their response as a function of energy and angle of incidence [2]; Measurement Uncertainty: A Practical Guide for Secondary Standards Dosimetry Laboratories, IAEA-TECDOC-1585, IAEA [3]; International Atomic Energy Agency. Radiation Oncology Physics: A Handbook For Teachers And Students, IAEA [4];] IAEA Safety Standards Series No. SSG-11 : Radiation Protection and Safety of Radiation Sources: International Basic Safety Standards, General Safety Requirements Part 3 [5].
Resource availability	NA

## Method details

### Background

Radiation monitoring of the working environment and its surrounding is an essential component of any good radiation protection program to guarantee that neither the operating staff nor the general public receive doses in excess of dose limits [1,6]. Thus, radiation protection monitoring instruments represent critical operational tools that meet radiation protection criteria; it is therefore essential to test their performance to ensure that they meet the required accuracy and the intended use necessitating their calibration. It is also a regulatory requirement for radiation-exposed workers to use only operational and tested control equipment when working with radioactive materials to ensure that radiation protection monitoring instruments can measure radiation dose with the accuracy required for their intended purpose [7,8]. In fact, if the dose of ionizing radiation absorbed by individuals at work is not controlled, people could face adverse health effects, specifically to their organs and DNA, depending on the duration radiation exposure, the distance from the source, the intensity of radiation and finally the sensitivity of the skin and exposed organs [9].

Radiation can be detected using various tools and procedures that rely on exposure or count rate readings. The choice of measuring instrument depends on the type of radiation and the specific measurement requirements.

Morocco employs a variety of ionizing radiation sources; hence any facility must be radiation-protected. Radiation protection includes both technical and cultural or ethical components, requiring not only the use of advanced technology to limit radiation exposure, but also the use of cultural or ethical standards to maintain radiation exposure as low as reasonably achievable (ALARA), with consideration of economic and societal factors [10]. What is considered a safe level of exposure is determined by ethical and cultural norms. What is acceptable is determined by a society's ideals rather than scientific risks. Fundamental values such as health and human dignity must be respected in radiation protective measures. Radiation risk is seen differently in different cultures. As a result, ethical and cultural norms ultimately frame radiation protection concepts and practices in a given community. Without agreed principles and norms, the technical aspect alone is insufficient.

Furthermore, Morocco adopted nuclear legislation, Law 142–12 [11], and established the Moroccan Agency for Nuclear and Radiological Safety and Security in compliance with this law, mandating personnel to use only operable and calibrated instruments in accordance with ISO 4037 [[2], [12], [13], [14]].

The aim of this work is to develop a digital graphical user interface to automate the determination of calibration factors for radiation protection measuring instruments. The Graphical user interface X-ray Gamma ray Calibration (XGC-GUI) was created using the python programming language which includes all the necessary libraries for the establishment of calibration factors, uncertainty measurement, calculation recording and electronic archiving of the calibration certificate in PDF format ported from PHP FPDF.

In this regard, representative results from the determination of the calibration factors for various radiation detectors and the uncertainty analysis performed at the Secondary Dosimetry Calibration Laboratory (SSDL), a member of the WHO/IAEA network within Morocco's National Center for Radiation Protection are also presented as an example.

### Calibration facilities

**Fig. 1:**
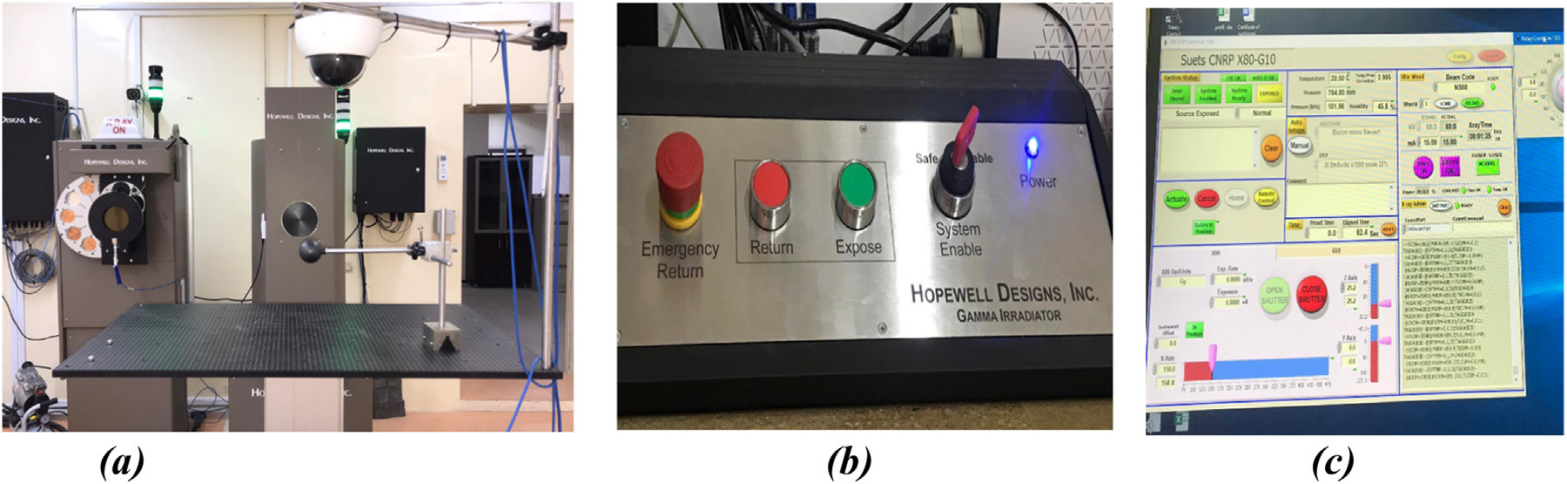
***(a)*** Gamma G10 irradiator assembly with automatic calibration bench. ***(b)****C*ontrol system. **(c)** Automated Irradiator Software.

The Fig. 1. ***(a)*** represents the G10–2–2600 model represented by Hopewell Designs [15] which is a dual source gamma beam irradiator with a capacity to hold both ^137^Cs (740 GBq +/- 20 %) and ^60^Co (74 GBq +/- 20%). The sources are double encapsulated and hermetically sealed in special stainless-steel shapes, driven by a stainless steel and tungsten rod. These sources, when combined with the attenuator set and the range of movement of the linear positioning track, provide a continuous range of exposure rates from 1 μSv/hr to 250 mSv/hr.

The rod moves inside a stainless-steel tube, sealed in the center of the shielded enclosure.

An additional tungsten shield surrounds the rod on both sides of the source to minimize leakage radiation.

Each source is moved pneumatically between its storage position and the irradiation point by means of a sensor capable of detecting the source position in less than a second.

The standard height of the beam centreline is 120 cm, and the overall size of the system is typically 60 cm wide by 40 cm deep by 150 cm height.

The System has a removable collimator to define the shape and size of the photon beam according to the ISO 4037 standard. The collimator provides a circular radiation beam ranging in diameter from 35 cm to 100 cm with a 15° angle, centered on the beam centreline.

A set of four lead attenuators can be combined or used individually to produce 16 beam intensity attenuation levels ranging from 0 to x8000. The four attenuators provide adjustments of x2, x4, x10, and x100. Each attenuator is actuated by a pneumatic cylinder.

The attenuator set is controlled by the automated irradiator Software shown in Fig. 1. ***(c).***

The linear positioning system is designed to precisely position radiation monitoring instruments at a specific distance from the gamma irradiator. The system can move up to four axes over a distance of 4 m with an accuracy of ± 1 mm. Standard movement ranges are: X axis 4 m; Y-axis 1 meter, and Z-axis 30 cm.

**Fig. 2:**
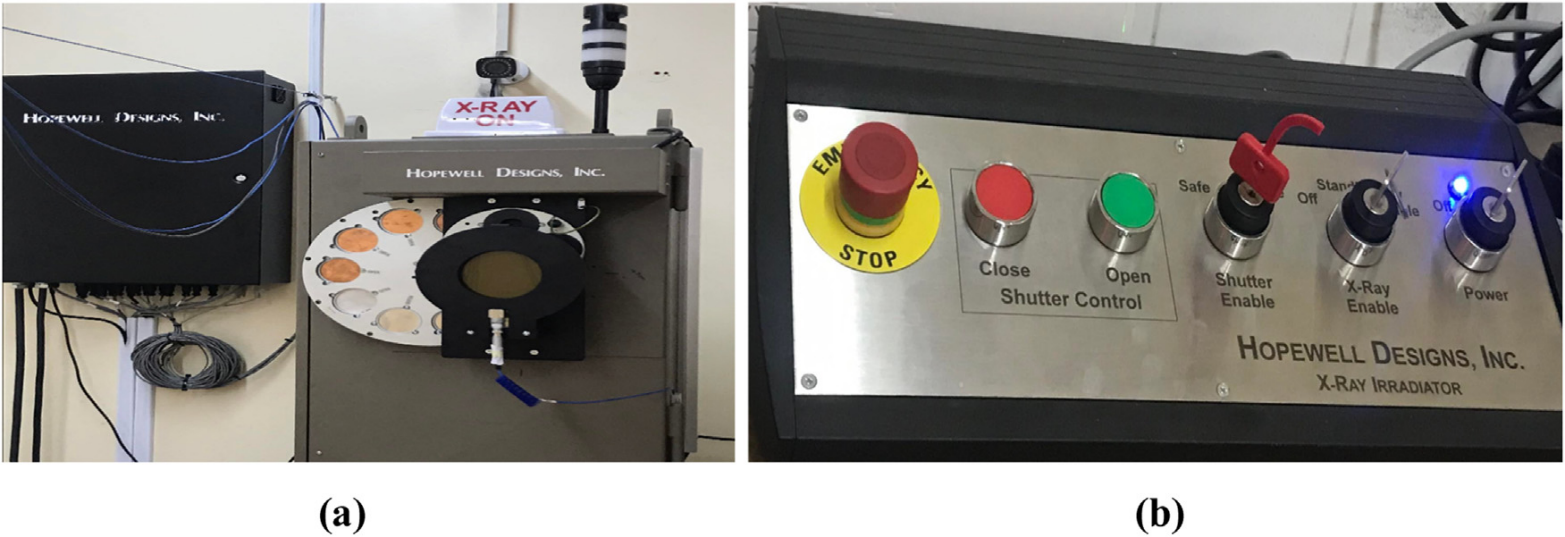
***(a)*** Model X80–320 kV X-ray beam irradiator used at SSDL. ***(b)****C*ontrol system.

The Fig. 2. ***(a)*** represent the X-ray irradiator Model X80–320 represented by Hopewell Designs [15] which is a complete system using X-rays to irradiate personnel dosimetry badges and radiation detection instruments in accordance with ISO-4037 requirements. It has a ceramic tube and a tungsten anode target with a 20° angle, a nominal focal spot, 3.0 mm small diameter, 5.5 mm large diameter and an inherent filtering of 3 mm of beryllium. The high voltage that may be applied to this X-ray tube varies from 15 to 320kVp, a current of 0.5 mA to 13 mA and a minimum power of 1500 W to 4200 W [16].

Furthermore, the Model Ap-3-M Aperture Assembly includes three replaceable lead apertures for adjusting the X-ray beam size based on the source-detector distance (SDD) and field size diameter (FSD) required for calibrating the measurement equipment. The apertures' dimensions are as follows:

- Source to detector distance (SSD) = 200 cm, Field Size Diameter (FSD) = 30 cm.
- SDD = 100 cm, FSD = 10 cm.
- For HVL measurements: SDD = 100 cm, FSD = 5 cm

Each aperture has a locking pin for easy installation and removal. When an aperture used, the diameter of the X-ray beam can be confined to the size indicated by the field configuration. This capability is critical for precise calibration of radiation measuring tools, assuring safety.

The charge generated by the X-ray and gamma-ray photons was measured using a spherical ionization chamber PS (50) TN 32007S and read from an associated Unidos Webline T10023 PTW electrometer by including the calibration factor assigned to each beam quality and radionuclide sources [9].

### Traceability chain

The secondary standard dosimetry system used is composed by a reference standard PTW ionizing chamber [17] model PS (50) TN32007 000007 calibrated at PTB laboratory associated with a PTW electrometer UNIDOS webline T10023 [18], and a working standard PTW ionization chamber model PS (50) TN32007S 000,007 calibrated at PTW laboratory associated with UNIDOS webline T10023.

### Calibration procedures

Calibration of survey meters is a regulatory requirement for radiation workers to use only functioning, pre-calibrated measuring instruments to ensure correct and valid radiation readings [19].

Calibration can be defined as a set of operations performed under specified conditions to establish the relationship between values indicated by a measuring instrument or system and the corresponding known true values of a quantity to be measured. In the field of radiation protection, the measuring instruments are usually area survey meters or personal dose and dose rate meters [20].

The calibration of area survey meters used for radiation protection purposes is based on 3 steps. Firstly, a reference instrument measures the air Kerma value at a calibration reference point in the radiation field and corrections are applied to this value to account for the effects of air temperature, air pressure, polarity and recombination. Secondly, an ISO 4037 Part 3 [12] conversion coefficient relating the physical quantity to the radiation protection quantity is used to establish the value of the relevant radiation protection quantity. Our laboratory has conducted a study [16] to evaluate and validate narrow-spectrum radiation characteristics in accordance with ISO 4037, including the calculation of conversion factors (*K_air_* to Hp (10)). This was done to ensure that we are using a matched radiation field in accordance with ISO 4037 and that the conversion coefficient given in the standard is valid for our spectra. By using conversion coefficients that are in conformity with ISO 4037, we can ensure that the radiation protection quantities we measure are accurate and reliable.

Finally, the calibrated instrument is put at this reference point to determine the instrument's response to the radiation protection quantity. A build-up plate of 3-mm polymethyl methacrylate (PMMA) shall be positioned in front of instrument when necessary to establish secondary electron equilibrium in the reference field according to ISO 4037–3 [2]. The modification of the radiation field by introducing the PMMA plate should be taken into account by multiplying the conversion coefficient with the correction factor k_PMMA_. The value of k_PMMA_ for ^137^Cs and ^60^Co is 1.

The calibration factor, CF, is defined as the conventional true value of the quantity H that the instrument is supposed to measure divided by the instrument's indicator measurement M:

(1) CF=H/M

The relative deviation of the instrument being calibrated from the reference ionization chamber must be within ± 30% of the expected dose rate. This requirement is defined by the laboratory according to IAEA recommendations [21] and Measurement Good Practice Guide No. 29 [22].

There are four calibration methods for survey meters described in IAEA safety reports series No. 16 [1]:

- Method 1 is to calibrate the instrument using a reference instrument without a monitor.
- Method 2 is to calibrate the instrument with a reference instrument and a monitor.
- Method 3 consists of calibrating the reference instrument and the instrument to be calibrated simultaneously by exposing them together to radiation.
- Method 4 involves calibrating the instrument in a known radiation field.

In our calibration facility calibrations are performed by the substitution method (method 4).

### Gamma-ray calibration method

Upon receiving survey meters, operational checks are performed, including battery, radiation response, and zero checks.

The steps of the calibration procedures for survey meters are as follows:

- Select the radioactive source.
- Choose the calibration point.
- Check that the reference standard is in the middle of the beam.
- Choose the attenuators that cover the survey meter measurement range.
- Measure of Kerma in the air *K_air_* in a reference field, using the reference standard.
- Record the display of the reference standard at least five times at regular intervals.
- Calculate the average value.
- Conversion of *K*_air__average measured value to H*(10) using conversion coefficients.
- Adjust the survey meter to the appropriate exposure/dose range.
- Place the survey meter in the calibrated source beam at an appropriate calibration distance from the radiation sources.
- Place the 3 mm PMMA build-up plate in front of the detector of the instrument to be calibrated.
- Check that the survey meter is in the middle of the beam.
- Background reading of survey meter (without beam).
- Expose the measuring instrument to radiation sources.
- Record the display of the measuring instrument at least five times at regular intervals.
- Calculate the average value, standard deviation and standard uncertainty of the readings obtained.
- Calculate calibration factors.

For various exposure or dosage ranges, repeat these procedures.

### X-ray calibration method

Upon receiving survey meters, operational checks are performed, including battery, radiation response, and zero checks.

The steps of the calibration procedures for survey meters are as follows:

- Select the appropriate beam quality based on energy range.
- Choose the calibration point.
- Check that the reference standard is in the middle of the beam.
- Choose the attenuators that cover the survey meter measurement range.
- Measure of Kerma in the air *Kair* in a reference field, using the reference standard.
- Record the display of the reference standard at least five times at regular intervals.
- Place the survey meter at the appropriate beam quality calibration distance.
- Turn on the survey meter and set it to the appropriate measurement range for the beam quality you are using.
- Measure the radiation at the calibration distance and record the value.
- Move the survey meter to a distance twice as far as the beam quality.
- Measure the radiation again at this new distance and record it.
- Repeat steps for distances three times, four times, five times, six times, and seven times the calibration distance, recording each measurement.
- Plot a graph of the distance versus the measured radiation for the survey meter (follows the inverse square law).
- Extrapolate the expected radiation value to the calibration distance.
- Calculate calibration factors.

Repeat these procedures for various radiation qualities.

### Uncertainty of measurement budget

Different sources of uncertainties are listed according to GUM [23], and the Practical Guide for Secondary Standards Dosimetry Laboratories [3]. Table 1, Table 2, Table 3.

**Table 1:** Characteristics of radionuclides used to calibrate gamma equipment.

Radionuclides	T_1/2_ /years	Energy/MeV
^60^Co	5.26	1.33/1.173
^137^Cs	30.17	0.662

**Table 2:** Radiation quality characteristics for different x-ray energy levels in accordance with ISO 4037 [16].

Radiation quality	Tube voltage (kV)	Additional filtration (mm)				1st HVL (mm)			2nd HVL (mm)		
		Pb	Sn	Cu	Al	ISO	SSDL	Deviation (%)	*ISO*	*SSDL*	*Deviation (%)*
N-30	30				4	1,16	1,13	2,65	1,28	1,24	3,13
N-40	40			0,21		0,085	0,082	3,66	0,093	0,092	1,08
N-60	60			0,6		0,234	0,24	2,5	0,263	0,26	1,14
N-80	80			2		0,578	0,6	3,67	0,622	0,62	0,32
N-100	100			5		1,09	1153	5,46	1,15	1205	4,78
N-120	120		1	5		1,67	1,76	5,11	1,73	1,85	6,94
N-150	150		2,5			2,3	2,4	4,17	2,41	2582	7,14
N-200	200	1	3	2		3,92	4,14	5,31	3,99	4,25	6,52
N-250	250	3	2			5,1	5,18	1,54	5,14	5,1	0,78
N-300	300	5	3			5,96	6253	4,69	6	6,37	6,17

**Table 3:** Characteristics of the air Kerma measurement chain for the gamma irradiator.

Reference Ionization chamber	[REF] TN32007	[SN] 000007
Electrometer	[REF] T10023	[SN] 000335
Measuring quantity	Kerma K_air_	
Detector calibration factor N_k_	5936.10^5^ Gy/C	
Electrometer calibration factor K_elec_	1 ± 0,5 %	
Beam	Correction factor k_Q_	Uncertainty (%)
^137^Cs	1	1,2
^60^Co	0,984	0,8
Working Ionization chamber	[REF] TN32007S	[SN] 000,007
Electrometer	[REF] T10023	[SN] 002,155
Detector calibration factor N_k_	5914.10^5^ Gy/C for ^137^Cs	
Detector calibration factor N_k_	5757 10^5^ Gy/C for ^60^Co	
Electrometer calibration factor K_elec_	1 ± 0,5 %	
Beam	Correction factor k_Q_	Uncertainty (%)
^137^Cs	1	2,5
^60^Co	0,996	2,5

The combined standard uncertainty of the calibration factor, CF(*k* = 2), was obtained by the quadratic sum of the two types of uncertainties, type A and Type B, multiplied by the student coefficient *k* = 2, for a 95% confidence level. The Table 5 lists the causes of uncertainty that were taken into consideration. The uncertainty of measurements with area monitors is typically within ±30% under standard laboratory conditions. The uncertainty for survey meter measurements will grow in the field [4].

### Program structure

*Unified Modelling Language UML (Use case diagram)*

**Fig. 3:**
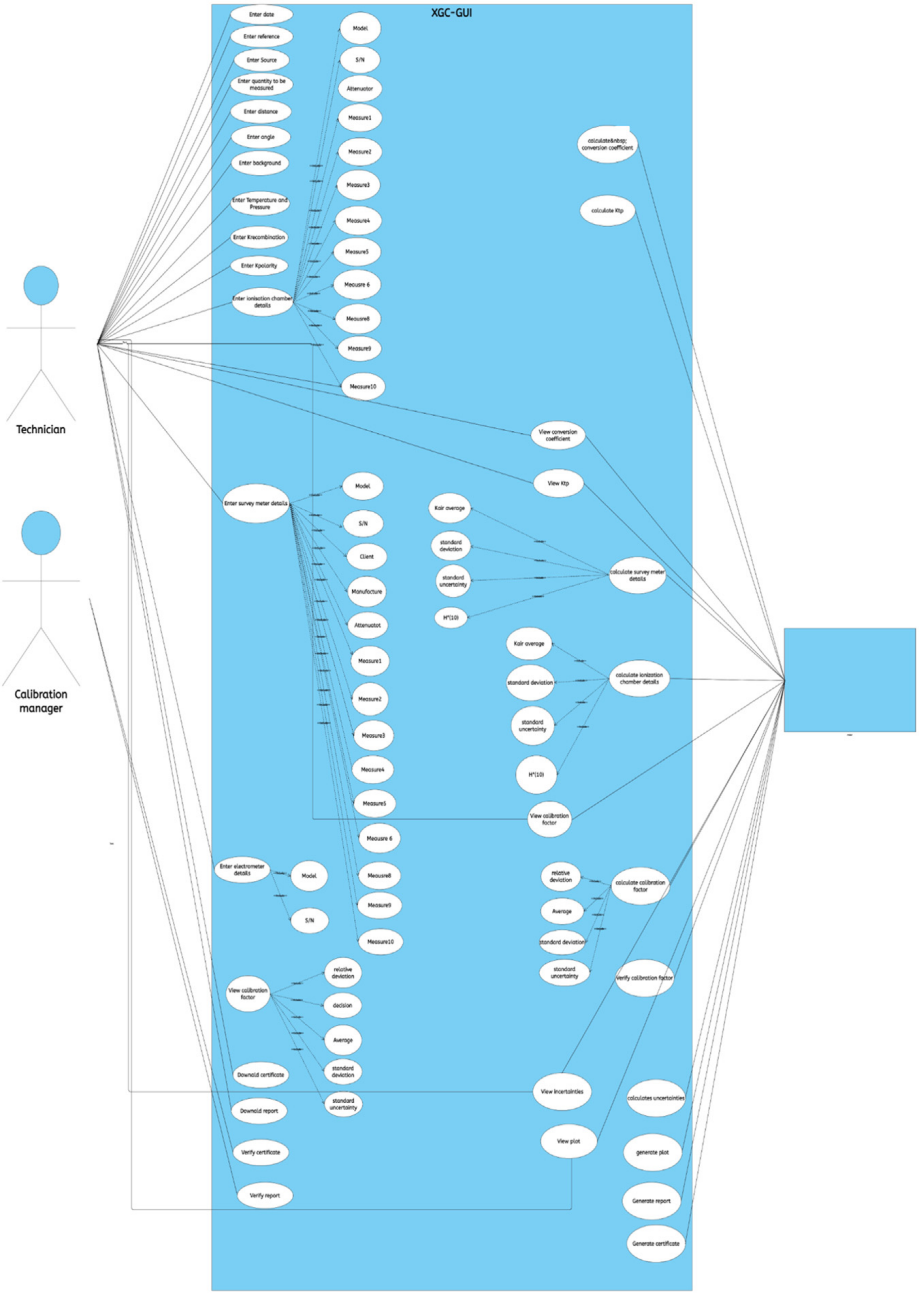
UML use case diagram of the graphical user interface developed.

Fig. 3 depicts the UML diagram, the application system, and the people and systems that interact with it. The system is represented by a rectangle and is known as XGC-GUI. This rectangle allows to define the scope of the system, everything within it happens inside the application, whilst everything outside of it does not happen in the application. Outside the rectangle are the actors represented by a man's stick figure. In our case, the technician and the calibration manager are the primary actors, and the secondary actor is the system itself that plays an active role in fulfilling the system's functionality by responding to the user's input by displaying the output results. The main actors must be on the left, and the secondary actor on the right. The oval shape represents an action that performs some kind of task in the system, it is inside the system since it presents actions that occur inside the application.

The actors are connected to the different features inside the box, indicating their interaction with the system. The use of the ``include'' keyword indicates that when a specific feature is executed, the included features must also be executed. For example, when entering the ``Enter ionization chamber details'' feature, it is necessary to provide the values for Meausure1 to Measure10.

Detailed explanation of some functionalities:

Actors:

- Technician: The Technician interacts with the survey meter calibration GUI application system. The user is responsible for initiating the various functionalities provided by the system.
- Calibration manager: verify and approve the calibration performed by the calibration system.
- System: The system is the secondary actor that represents the software or environment in which the survey meter calibration GUI application operates.

Some functionalities:

- ``Enter date'': allows the user to enter the date on which the calibration was carried out in order to guarantee the traceability of the calibration.
- ``Enter reference'': allows the user to enter the reference of the calibration certificate and report generated at the end of the calibration in order to store this information for future use.
- "Enter source": allows the user to enter the radiation source allowed for calibration to ensure that the radiation source is appropriate for the instrument being calibrated and for the specific application.
- "Enter the quantity to be measured": allows the user to enter the physical quantity to be measured, generally it is H*(10).
- ``Enter distance'': allows the user to enter the distance between the radiation source and the calibrated instrument to ensure that the radiation source is placed at an appropriate distance from the calibrated instrument and at the same distance of reference instrument.
- ``Enter angle'': allows the user to enter the angle between the radiation source and the calibrated instrument. This information is important to ensure that the radiation source is placed at an appropriate angle to the calibrated instrument.
- ``View conversion coefficient'': allows the user to view the conversion coefficient on the system's graphical interface. The conversion coefficient is calculated from the information entered in the "Enter the quantity to be measured", "Enter the distance" and "Enter the angle" functions.
- ``Enter temperature and pressure'': allows the user to enter the ambient temperature and pressure at the time of calibration. This information is important to ensure that ambient conditions are taken into account when calculating the calibration factor.
- ``View k_TP_'': allows the user to view the k_TP_ value, which is calculated from the information entered in the ``Enter temperature and pressure'' functionality. *k_TP_* is used to correct measurements for ambient temperature and pressure.
- ``Enter background'': allows the user to enter the background value of the instrument to be calibrated. This value is used to subtract the background noise from the measurements made with the instrument to be calibrated in order to take into account the effect of the background noise on the measurements.
- ``Enter Krecombination'' and ``Enter Kpolarity'': These functionalities allow the user to enter the values of *Krecombination* and *Kpolarity* to make the necessary corrections in the *Kair* determination phase. These values are correction factors that are used to account for recombination and polarity effects.
- ``Enter Ionization Chamber Details'': allows the user to enter the values for Measure1 to Measure10, the model, S/N and the attenuators which are the details of the ion chamber used for calibration.
- ``Verify Calibration Factor'': This feature allows the system to verify the calibration factor using the ion chamber details and survey meter details entered previously.
- ``Generate Report'': allows the system to generate a calibration report that summarizes the details of the calibration performed, including the results obtained and the values measured.
- ``Generate Certificate'': allows the system to generate a calibration certificate that certifies that the survey meter has been correctly calibrated to specified standards.

### Programming step and libraries

The development process for the XGC-GUI involved four steps, starting with the insertion of interface components such as buttons, labels, and tables on a window using PyQt5.

The second step involved the creation of a file based on event-driven programming, where functions are called in response to user-triggered events. These functions were defined to perform specific actions, such as opening files, saving data, or updating the interface. This programming paradigm enables efficient and responsive interaction with the GUI.

The third and fourth steps involved the coding of two files to generate reports and calibration certificates. This was achieved using the PHP FPDF library, which is a popular library for generating PDF files in PHP. The FPDF library provides a range of functions for creating text, graphics, and tables in PDF documents, which can be customized according to the specific needs of the application.

The libraries used in the XGC-GUI development process are explained below:

- Tkinter: a Python library for designing graphical user interfaces. It includes a collection of widgets, such as buttons and labels, that may be used to create interactive applications.
- sys: a library that provides access to some variables and functions used or maintained by the Python interpreter
- Colorama: a library for enhancing console output with color.
- PyQt5: a library used to build desktop programs with graphical user interfaces. It is built on the Qt framework and includes a large choice of widgets and tools for developing interactive applications.
- Numpy: a library for numerical computing. It includes a set of functions for working with arrays, matrices, and other numerical data, making complex mathematical operations simple.
- Statistics: a library for performing statistical calculations in Python.
- Math: a library for mathematical operations in Python
- operator: a library for performing operations on Python objects, such as addition, subtraction, and division
- PySerial: establish a serial connection with the electrometer and retrieve its values.
- FPDF: FPDF a library used for generating PDF files. It offers a range of functions for creating text, graphics, and tables in PDF documents.

## Results

**Fig. 4:**
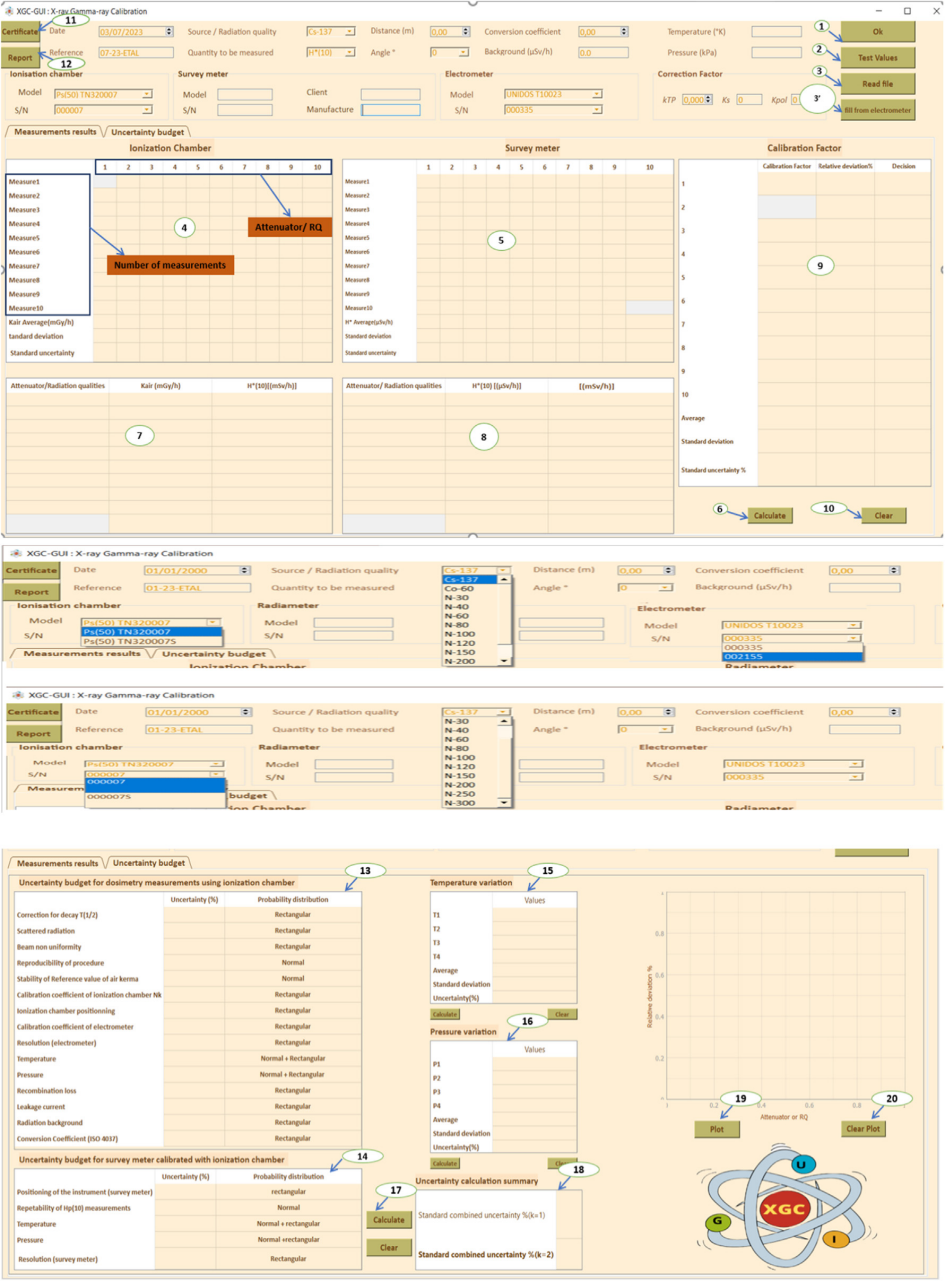
XGC-GUI graphical user interface for radiation monitoring instrument calibration: initial view.

Fig. 4 shows an initial view of the XGC-GUI for calibration of radiation monitoring instruments where:

1. Button to calculate conversion coefficient based on ISO 4037 standard and user-selected values for source/radiation qualities, quantity to be measured, distance, and angle using the comboboxes available in the UI, as well as calculate correction factor k_TP_ based on manually entered temperature and pressure values. Noted that the value of k_TP_ can also be automatically transported from the system (Fig. 1.c).
2. Button to randomly fill in Tables 4 and 5 for GUI testing purposes.

**Table 4:** Characteristics of the air Kerma measurement chain for the Xray irradiator.

Reference ionization chamber	[REF] TN32007	[SN] 000007
Electrometer	[REF] T10023	[SN]000335
Measuring quantity	Air Kerma K_air_	
Detector calibration factor N_k_	5936.10^5^ Gy/C	
Electrometer calibration factor K_elec_	1 ± 0,5%	
Beam	Correction factor k_Q_	Uncertainty (%)
N-60	1,198	0,8
N-80	1,165	0,8
N-100	1,085	0,8
N-120	1,038	0,8
N-150	1,016	0,8
N-200	1	0,8
N-250	1,002	0,8
N-300	1,006	0,8
Working ionization chamber	[REF] TN32007S	[SN] 000,007
Electrometer	[REF] T10023	[SN] 002,155
Measuring quantity	Air Kerma K_air_	
Detector calibration factor N_k_	6398.10^5^ Gy/C	
Electrometer calibration factor K_elec_	1 ± 0,5%	
Beam	Correction factor k_Q_	Uncertainty (%)
N-20	1,471	2,5
N-30	1,104	2,5
N-40	1,080	2,5
N-60	1,124	2,5
N-80	1,087	2,5
N-100	1	2,5
N-150	0,935	2,5
N-200	0,910	2,5
N-250	0,915	2,5

**Table 5:** Typical uncertainty budget.

	Sources of uncertainty	Type
Ionization chamber	Decay Correction	type B
	Scattered radiation	type B
	Beam non uniformity	type B
	Reproducibility of procedure	type A
	Stability of reference value of air Kerma	type A
	Calibration coefficient of ionization chamber Nk	Type B
	Ionization chamber positioning	Type B
	Calibration coefficient of electrometer	Type B
	Resolution (electrometer)	Type *A* +Type B
	Temperature	Type *A*+ Type B
	Pressure	Type B
	Recombination loss	Type B
	Leakage current	Type B
	Radiation background	Type B
	Conversion coefficient (ISO 4037)	Type B
Survey meter	Positioning of the instrument	Type B
	Repeatability of Hp (10) measurements	Type A
	Temperature	Type *A*+ Type B
	Pressure	Type *A* + Type B
	Resolution (survey meter)	Type B
3. Button to read *K_air_* data from text file and populate Table 4 with measured values (adjusted for background) from ionization chamber. 3′. Button to automatically transmit *K_air_* from electrometer and fill Table 4.
4. Table 4 for filling in *K_air_* values measured by ionization chamber and read by electrometer. The values can be entered either manually or imported from a .txt file using button 3 or directly from the electrometer by using pySerial library and timeout module using button 3′.
5. Table 5 for filling in H*(10) values read by instrument to be calibrated.
6. Button to fill in Tables 7, 8, and 9.
7. Table 7 for connecting physical quantities with radiation protection quantities, including attenuator/radiation qualities entered manually based on the control irradiator software, *K_air_* values measured by ionization chamber, and H*(10) values corrected for k*_TP_* (Temperature & pressure correction factor), *k_s_* (recombination correction factor), and *k_pol_* (polarity correction factor).
8. Table 8 for recording measurements of instrument to be calibrated, including attenuators/beam code used, average H*(10) value in µSv/h, and H*(10) value in mSv/h.
9. Table 9 for calculating calibration factor (*N_k_*) for instrument based on values in Tables 7 and 8, using the equation provided.
  (2) $$\text{Nk}=\;\frac{H*\left(10\right){\;}_{ionization\;chamber}}{H*\left(10\right){\;}_{instrument\;to\;be\;calibrated}}$$

The relative deviation of the instrument being calibrated from the reference ionization chamber is calculated using the formula:

(3) $$\frac{H*\left(10\right){\;}_{ionization\;chamber}-H*\left(10\right){\;}_{instrument\;to\;be\;calibrated}}{H*\left(10\right){\;}_{ionization\;chamber}}$$

Each observed measurement should be within ± 30% of the expected dose rate.

10. Button to delete the content of the tables.
11. Button to display the calibration certificate, showing data in line with current standard criteria.
12. Button to display a report containing all input and output data.
13. Table for measuring uncertainty of the measurement budget.
14. Table for measuring uncertainty of the survey meter.
15. Table for determining variation of temperature for evaluation of type A uncertainty due to temperature effect.
16. Table for determining variation of pressure for evaluation of type A uncertainty due to pressure effect.
17. Button to complete Table 18 and calculate standard combined uncertainty (square-root of the linear sum of squared standard uncertainty components).
18. Button to plot the Calibration Factor CF as a function of attenuator or RQ (Radiation quality).
19. Button to erase the plot*.*

### Calculation example

**Fig. 5:**
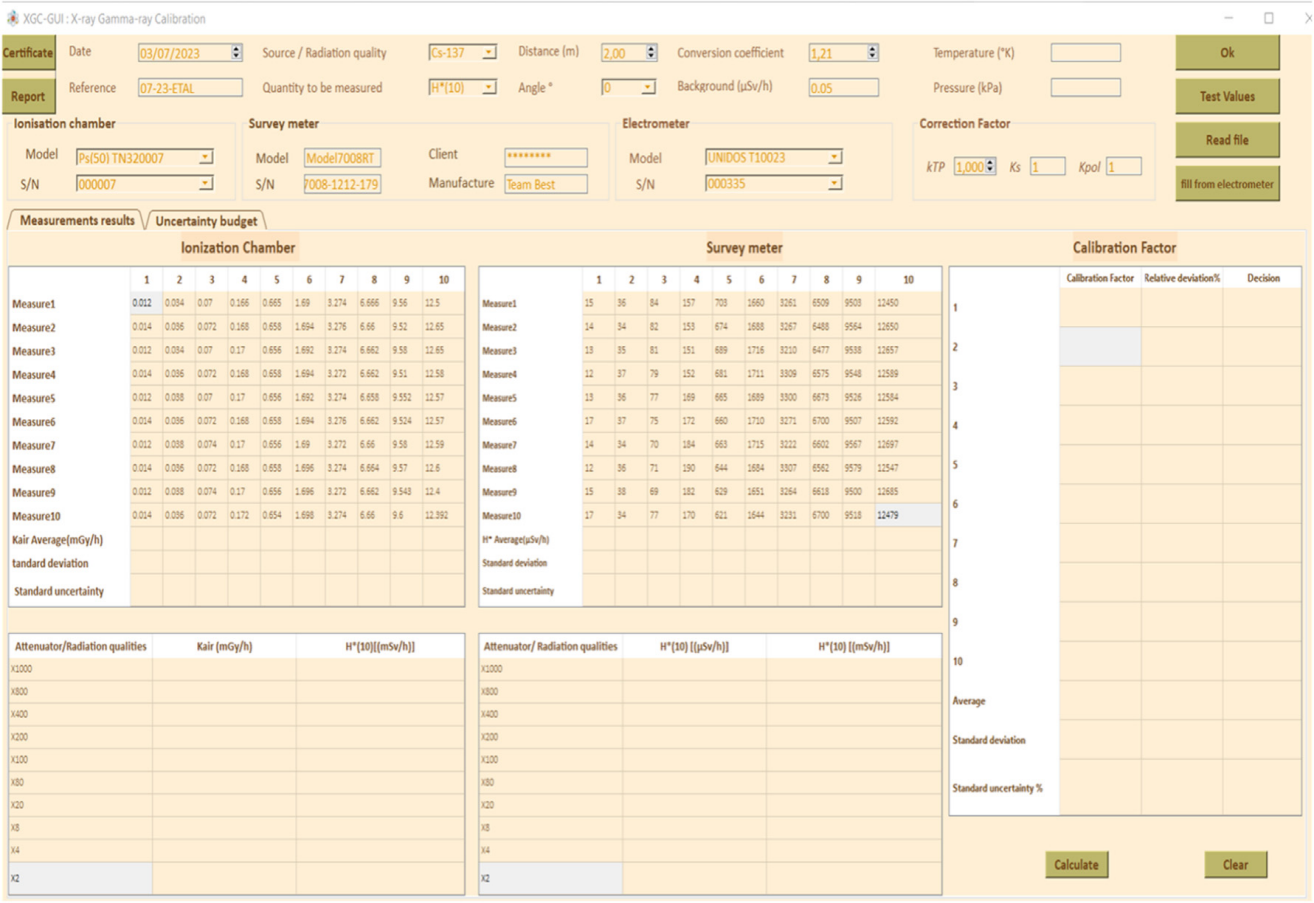
Input data: calibration factor calculation.

**Fig. 6:**
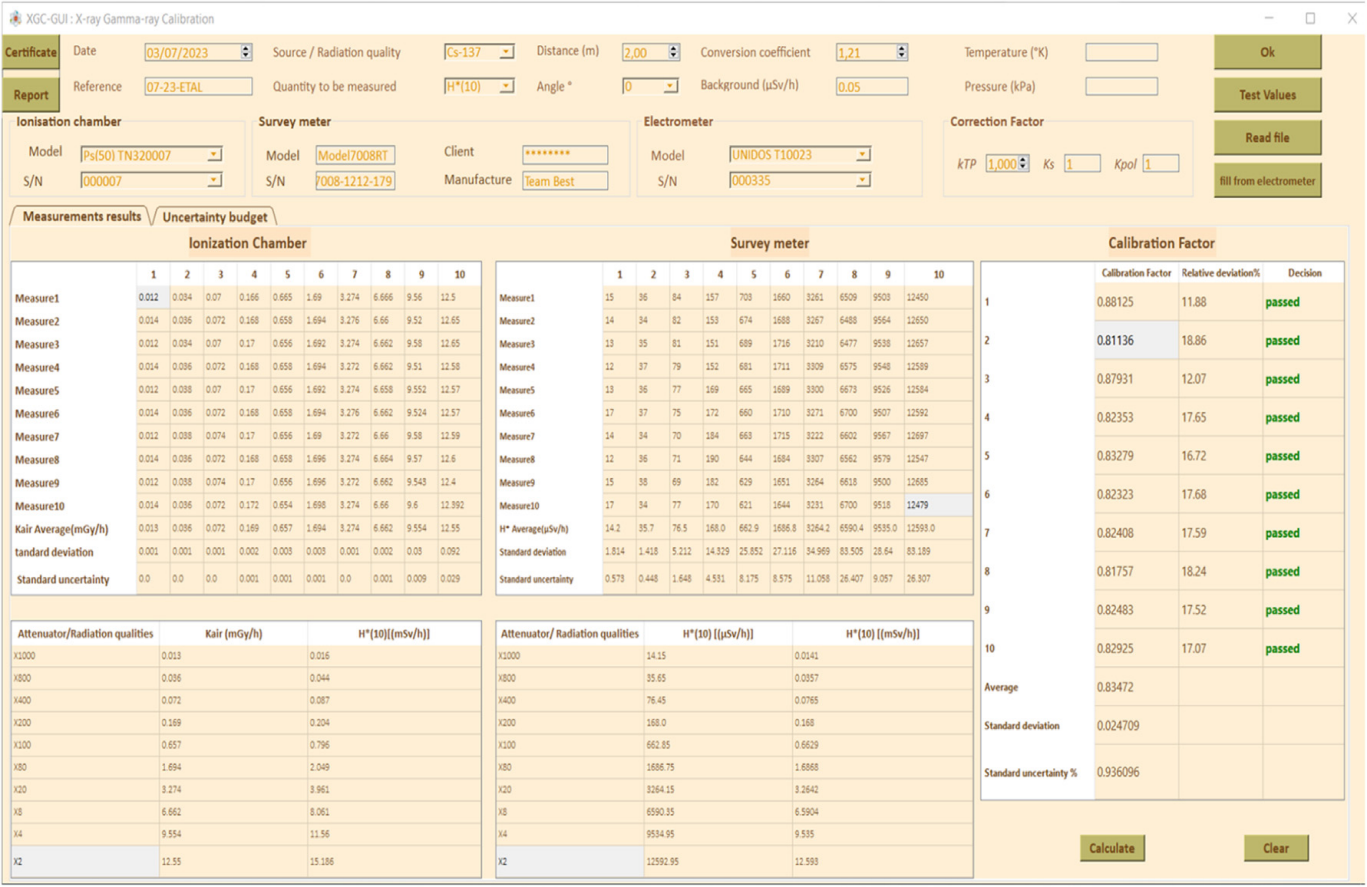
Output data: calibration factor calculation.

**Fig. 7:**
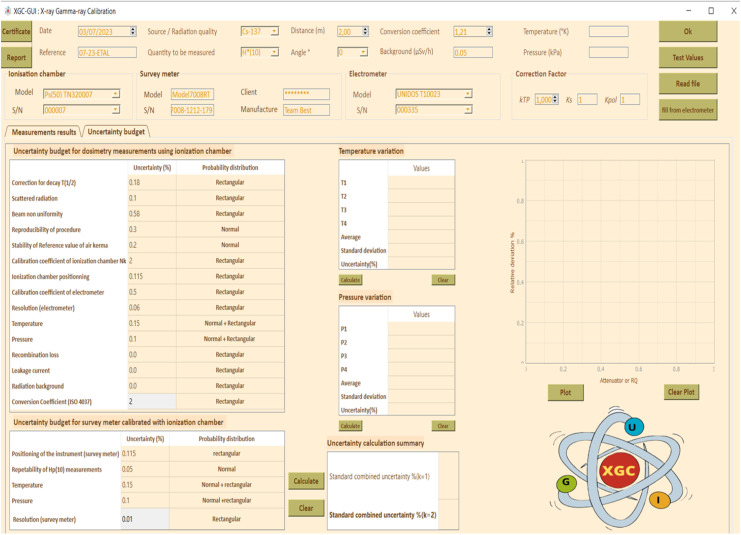
Input data: uncertainty calculation.

**Fig. 8:**
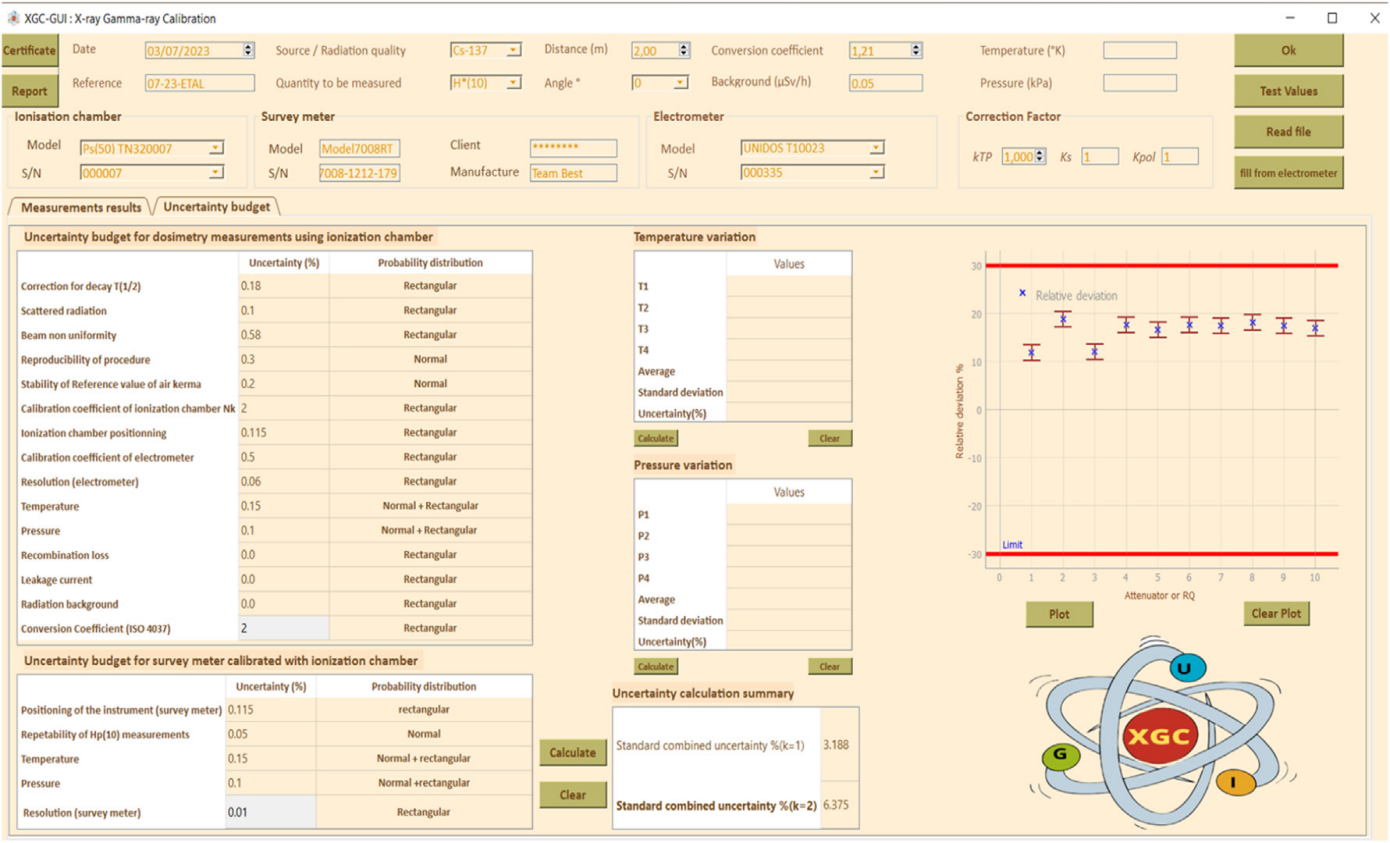
Output data: uncertainty analysis.

**Fig. 9:**
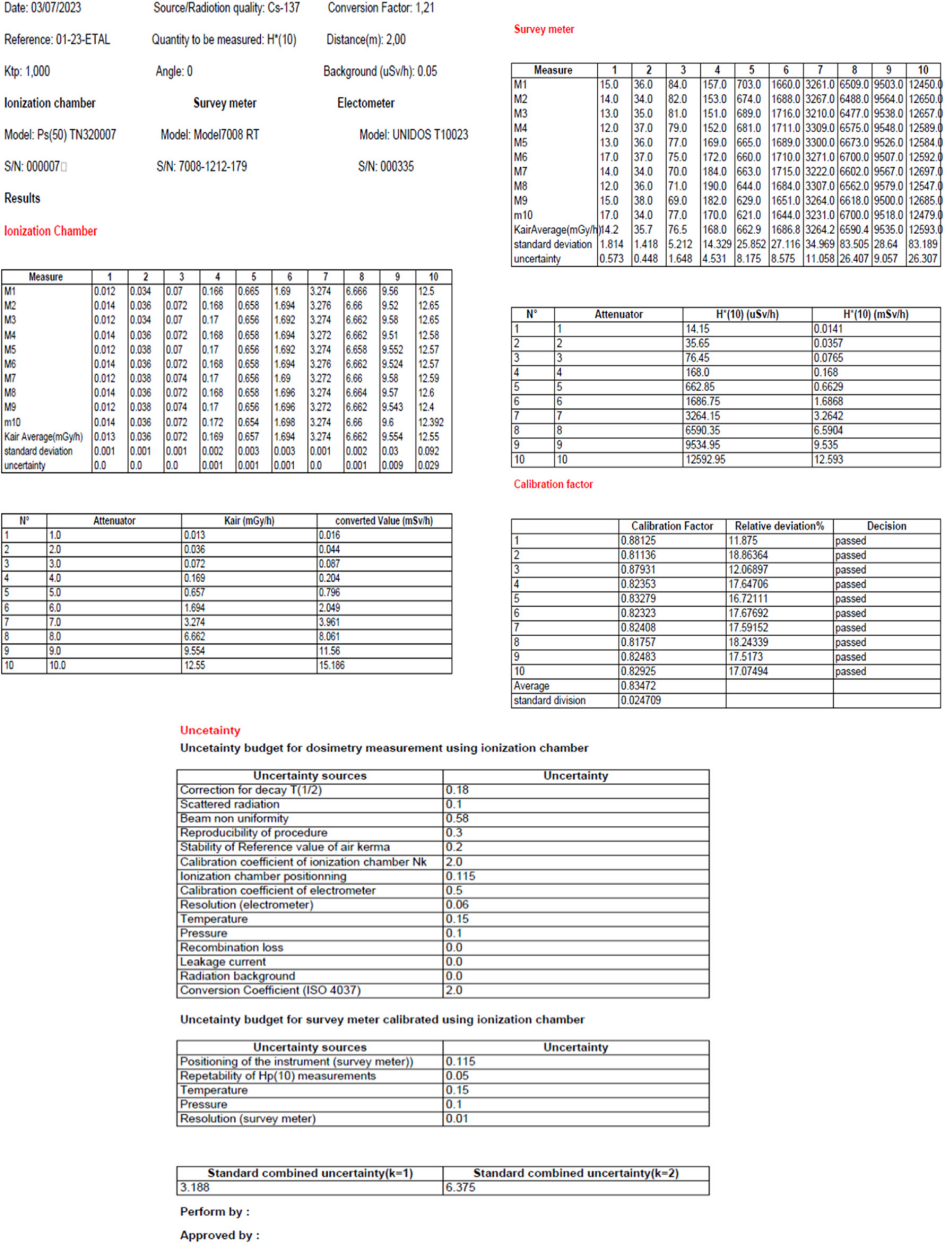
Report output: raw measurement data.

**Fig. 10:**
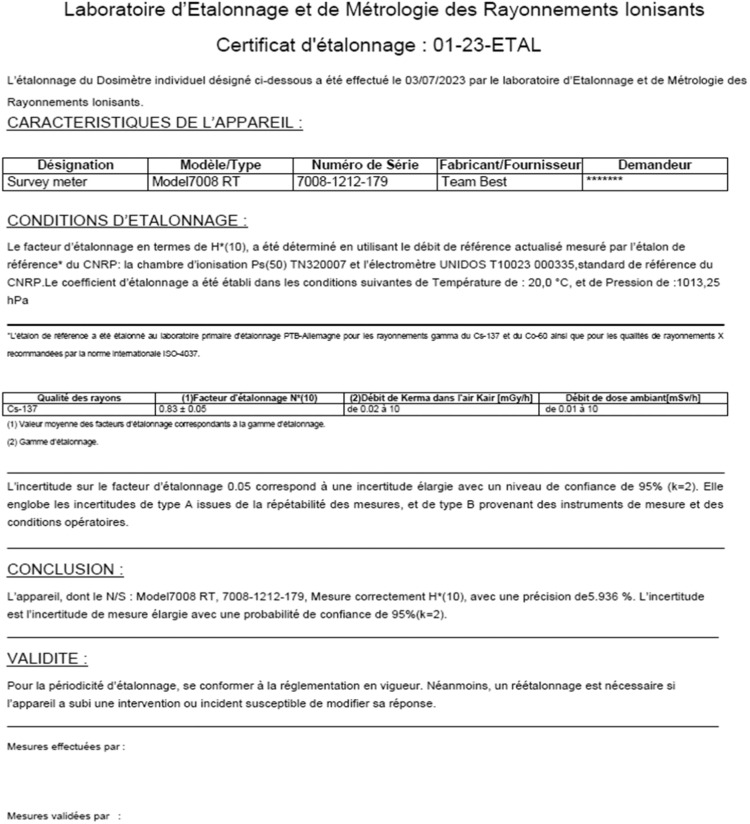
Calibration certificate.

Fig. 5 through10 illustrate the importance of following standards (ISO 4037, ISO 17,025, and IAEA recommendations [[1], [2], [5], [12], [13], [14], [24], [25]] to ensure accurate and reliable measurement results. The input and output data shown in these figures are necessary for the calculation of the calibration factor and associated uncertainty, which are key elements in ensuring accurate and reliable measurements. Fig. 6, Fig. 7, Fig. 8, Fig. 9, Fig. 10.

Fig. 9 also shows the importance of following ISO 4037 standards and IAEA recommendations by recording the raw measurement data to ensure traceability of the measurements made. Finally, Fig. 11 shows the requirement of ISO 4037 and the IAEA recommendations to provide a calibration certificate to client to ensure compliance with industry specifications and traceability of measurements made.

Based on the found results, the combined standard uncertainty of the calibration factor was found to be 6.3% for a student coefficient *k* = 2, which is within the requirement of being less than or equal to 30%. Additionally, the uncertainty due to the standard deviation was found to be 0.93%.

Overall, these results indicate that the calibration process was conducted with an acceptable level of uncertainty according to the ISO 4037 standard and the IAEA recommendation.

To summarize, ISO 4037 and the IAEA recommendations are essential international standards for ensuring the quality and reliability of measurements made with radiation protection photon measurement instruments and for providing client confidence in the measurement results provided by laboratories.

## Conclusion

Overall, the study highlights the importance of adhering to international standards to ensure the quality and reliability of measurements made with photon measurement instruments in radiation protection. The development of a digital graphical user interface can help laboratories meet these standards more efficiently and provide customers with reliable and accurate measurement results [26].

In summary, by using a digital graphical user interface and electronic archiving that meets current standards and regulations, the calibration of radiation protection measuring devices may be enhanced, mistakes reduced, and readings more precisely tracked. These technical developments help to increase the accuracy and dependability of measurement findings while reducing their negative environmental effects and offering a safer and more effective method of data storage.

## Ethics statements

Not applicable

## Data availability statement

No data was used for the research described in the article.

## CRediT authorship contribution statement

**Omaima Essaad Belhaj:** Writing – review & editing, Writing – original draft, Methodology, Formal analysis. **Siham Belhaj:** Visualization, Validation, Methodology. **Meryeme Bellahsaouia:** Visualization, Methodology. **Younes Sadeq:** Visualization, Methodology. **Maryam Hadouachi:** Visualization, Methodology. **Khaoula Laazouzi:** Visualization, Methodology. **Assia Arectout:** Visualization, Methodology. **Hamid Boukhal:** Visualization, Validation, Supervision. **Chakir El mahjoub:** Visualization, Investigation. **Tahar El Khoukhi:** Writing – review & editing.

## Declaration of Competing Interest

The authors declare that they have no known competing financial interests or personal relationships that could have appeared to influence the work reported in this paper.

## Data Availability

No data was used for the research described in the article.
